# Ventilation distribution in rats: Part I - The effect of gas composition as measured with electrical impedance tomography

**DOI:** 10.1186/1475-925X-11-64

**Published:** 2012-09-04

**Authors:** Kimble R Dunster, Marlies Friese, John F Fraser, Gary J Cowin, Andreas Schibler

**Affiliations:** 1 Paediatric Critical Care Research Group, Paediatric Intensive Care Unit, Mater Children’s Hospital, South Brisbane, QLD, Australia; 2 Critical Care Research Group, The Prince Charles Hospital, Brisbane, QLD, Australia; 3 Centre for Advanced Imaging, The University of Queensland, Brisbane, QLD, Australia; 4 Medical Engineering, Queensland University of Technology, Brisbane, QLD, Australia; 5 Paediatric Intensive Care Unit, Mater Children’s Hospital, South Brisbane, 4101, QLD, Australia

**Keywords:** Electrical impedance tomography, Ventilation distribution

## Abstract

**Abstract:**

The measurement of ventilation distribution is currently performed using inhaled tracer gases for multiple breath inhalation studies or imaging techniques to quantify spatial gas distribution. Most tracer gases used for these studies have properties different from that of air. The effect of gas density on regional ventilation distribution has not been studied. This study aimed to measure the effect of gas density on regional ventilation distribution.

**Methods:**

Ventilation distribution was measured in seven rats using electrical impedance tomography (EIT) in supine, prone, left and right lateral positions while being mechanically ventilated with either air, heliox (30% oxygen, 70% helium) or sulfur hexafluoride (20% SF_6_, 20% oxygen, 60% air). The effect of gas density on regional ventilation distribution was assessed.

**Results:**

Gas density did not impact on regional ventilation distribution. The non-dependent lung was better ventilated in all four body positions. Gas density had no further impact on regional filling characteristics. The filling characteristics followed an anatomical pattern with the anterior and left lung showing a greater impedance change during the initial phase of the inspiration.

**Conclusion:**

It was shown that gas density did not impact on convection dependent ventilation distribution in rats measured with EIT.

## Introduction

Regional ventilation distribution in human and animal studies is currently determined by measuring the distribution of an inhaled tracer gas. Pulmonary function tests such as multiple breath inert gas washout can measure global ventilation inhomogeneity but cannot localize regional ventilation distribution [1]. Radio-labeled ventilation scanning or newer imaging techniques such as computed tomography (CT) and magnetic resonance imaging (MRI) using hyperpolarised helium (He3 MRI) are used to investigate regional ventilation distribution [2-4]. He3 MRI uses pure helium, which has a density and viscosity significantly different from the gases normally breathed spontaneously or used in mechanical ventilation. The effect of gas density on pulmonary compliance [5], the mechanics of breathing and pulmonary gas exchange [6,7] has been investigated in the past but no study has addressed the impact of gas density on regional ventilation distribution. Inhaled tracer gases with different gas density (i.e. helium and SF_6_) are used to measure ventilation distribution in lung disease such as cystic fibrosis [8,9]. It has been shown that the location of the convection diffusion front is dependent on gas density and also on the underlying disease process [10]. In order to investigate the impact of gas density on regional ventilation distribution per se, a measurement technique independent of gas composition and density must be used. Electrical impedance tomography (EIT) measures transthoracic impedance changes during breathing and images based on the change of gas volume within the chest are obtained. Since the commonly used tracer gases such as helium and sulfur hexafluoride (SF_6_) have negligible electrical conductivity, as does air, EIT assessment of regional ventilation distribution is independent of gas composition and density [8].

The rationale of Part I of the current study was to investigate whether density and diffusion capacity of an inhaled gas per se impacts on regional ventilation distribution, global ventilation inhomogeneity or the filling characteristics of the lung. If gas density/diffusion capacity is not a major determinant of the measured ventilation inequalities then a comparison of the EIT technology with hyperpolarized He3 MRI imaging is valid, which is describe in Part II.

## Methods

### Study design

Using EIT, ventilation distribution and regional lung filling were measured in seven rats in each of four body positions and with three gas mixtures of differing density randomly inhaled.

### Animal preparation

Animal ethics approval was obtained from The University of Queensland. Seven Wistar rats (8 to 10 weeks of age, 279 ± 36 g of either sex) were studied. The rats were anaesthetized, intubated and prepared accordingly to our standard protocol [11]. Three gas mixtures were used, in random order, to ventilate the rat – air (ρ = 1.2 gL^-1^, viscosity: 186 μP); -, 70% helium with 30% oxygen (Heliox, ρ = 0.51 gL^-1^, viscosity: 199 μP); - SF_6_, 20% sulphur hexafluoride with 20% oxygen and 60% air (ρ = 2.4 gL^-1^, viscosity: 270 μP (calculated)). All gas mixtures had compressibility factors within 1% ( http://encyclopedia.airliquide.com/encyclopedia.asp). With each gas mixture, the rat was randomly placed in each of four postures: prone, supine, left- and right-lateral and ventilated using a time-cycled, pressure-limited ventilator based on that of Hedlund [12] with a respiratory rate of 80 breaths per minute and a tidal volume of ~10 mL/kg.

### Electrical impedance tomography (EIT)

A Göttingen GoeMF II EIT tomograph (Sensormedics/ VIASYS Healthcare, Netherlands) was used [13]. The basic principles of EIT have been published elsewhere [14,15]. The rats were circumferentially shaved around the chest and 16 epicardial pacing wires (Medtronic Inc, Minneapolis, MN, USA) were sutured in an equidistant fashion through the skin and the panniculosus carnosus [11]. EIT measurements were made with a 100 kHz injected current at 44 images per second. A sensitivity-weighted back-projection algorithm [16] was used to reconstruct a 32×32 pixel image of the distribution of relative impedance changes. A minimal data set length of 60 second or at least 60 breaths for analysis were required.

### Data analysis

Functional EIT data was analysed offline using custom developed software (MATLAB, Mathworks, 7.2, Natick, MA, USA). Data were filtered using a band pass filter including the first and second harmonic of the respiratory rate [17-19]. With this filter in place, the ventilated regions were defined as regions in which the impedance signal was greater than 20% of the peak impedance signal [20,21].

### Ventilation distribution (VD)

Three measures of VD were employed – the amplitudes of regional impedance change, the geometric centre (GC) as a measure of regional VD and the global inhomogeneity index (GI) as a measure of global VD.

Amplitudes of regional impedance changes for the anterior, posterior, right and left side of the ventilated regions were calculated by averaging the end expiratory to end inspiratory impedance differences for each pixel in the region of interest (ROI) [22]. To account for the unequal number of pixels analysed in the different ROIs, the average amplitude of each ROI was reported.

The GC of the EIT image was calculated for the entire image [23]. The GC defines the centre of ventilation using a balanced averaging of pixel values from right to left and from anterior to posterior.

The GI quantifies the tidal volume distribution within the ventilated region [24]. A median value for all pixel amplitudes was calculated across the entire image and the sum of the absolute difference between the median and every pixel indicated the tidal volume distribution in the ventilated region. The value was then normalised to the number of pixels included. The lower the GI value the more homogenous the ventilation is distributed.

### Regional (temporal) filling characteristics

The filling index describes the rate of volume change between different ventilated regions. In theory, gas density should impact on the temporal filling of lung regions with the rate of volume change in the dependent lung increased with more dense inhaled gas. Regional volume change was compared to global volume change by plotting the impedance change of the relevant region against that of the global signal to form a curve [25]. The slope (g) was fitted, using a Levenberg-Marquardt method, to the following equation [25]:

(1) $$I\left(g\right)=a\cdot g^{\mathrm{FI}}+c$$

where *I(g)* is the regional impedance change, g is the global impedance change, *FI* is the regional filling index and *a* and *c* are constants.

The filling index, *FI*, describes the shape of the curve. A linear relationship (FI = 1) is found if the rate of volume change of a ROI is the same as the global lung. If the rate of change in a ROI is initially less but increases as inspiration continues, the curve has a concave shape (FI > 1). If the rate in a ROI during the initial phase of the inspiration is greater than the global lung but decreases towards the end, then the curve has a convex shape (FI < 1).

### Statistics

A general linear model was used to seek interactions between position or gas composition on measured parameters. Results were described using the mean and confidence intervals. An ANOVA with Bonferoni for repeated measurements was used to compare parameters within each position. Data was described using mean and standard error of the mean. For statistical analysis SPSS version 15.0 (SPSS Inc., Chicago, IL) was used. Significance was accepted at p < 0.05.

## Results

### Regional (spatial) ventilation distribution

**Figure 1:**
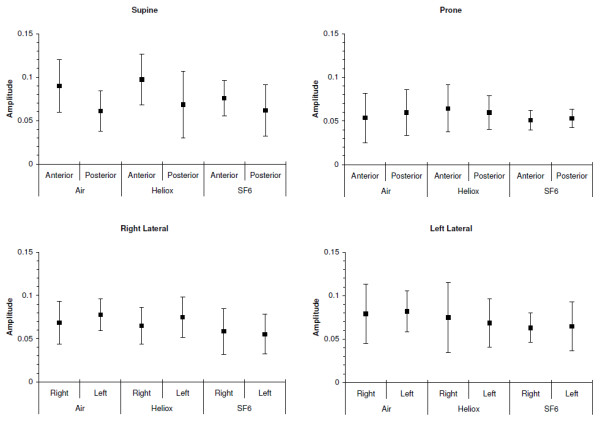
**Magnitude of regional impedance amplitudes using air, heliox and sulfur-hexafluoride (SF**_**6**_**)**.

The gas mixture had no effect on the average amplitude of impedance changes (*P* = ns) (Figure 1). In supine position there was a trend toward better ventilation in the anterior lung, whereas in prone no such trend was observed. In general, the non-dependent lung was better ventilated than the dependent lung in supine or prone positions except for prone position and using Heliox, the dependent lung was slight better ventilated. No differences for the average amplitudes between the right and the left lung in lateral position were found.

**Figure 2:**
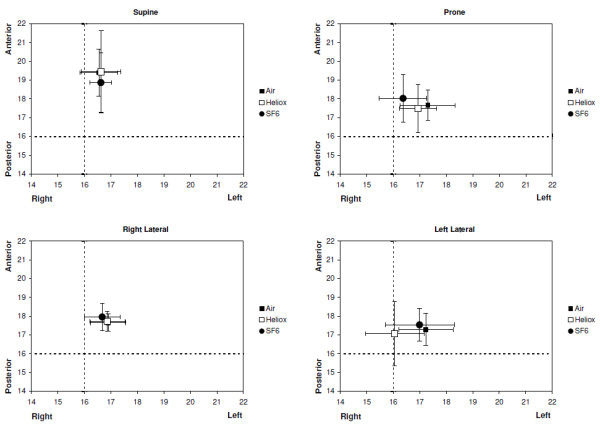
**Geometric centres (mean and 95% confidence interval) for the three gas mixtures in each of four body positions.** The position of the GC is located on the EIT image using the 32x32 pixel matrix. The dashed lines indicate the centre of the image.

Gas composition did not affect the location of the GC (*P* = ns) in any position (Figure 2). Body position however did impact on the location of the GC with the GC in supine significantly more located in the anterior lung than in prone (*P* < 0.05). In both, right and left lateral position the GC remained unchanged (*P* = ns).

**Figure 3:**
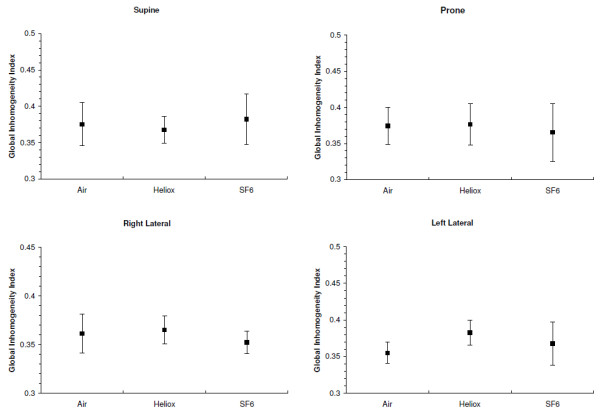
Global Inhomogeneity indices (mean and 95% confidence interval) for the three gas mixtures in each of four body positions.

The values of the GI for all three gas mixtures and body positions are shown in Figure 3. There was no interaction between position and gas density and the GI was similar for all positions and gas mixtures (*P* = ns).

### Regional (temporal) filling characteristics

**Figure 4:**
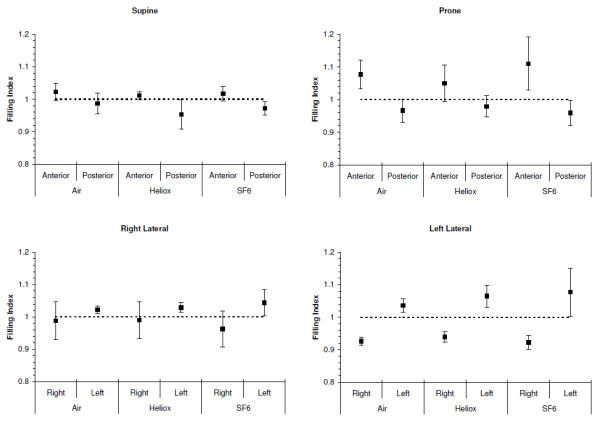
**Filling indices for the three gas mixtures in each of four body positions.** The FI for the anterior lung was significantly different to the posterior lung for supine and prone position (P < 0.001). Similarly the FI of the left lung was significantly different to the right lung in both the right and left lateral position (P < 0.001). The mean and 95% confidence interval are presented. The dotted line indicates an FI = 1, where the rate of impedance change in the ROI is similar to the global impedance change. # indicates significant differences (P < 0.001) between anterior and posterior, and between left and right respectively.

Filling indices are shown in Figure 4. No interaction between position and gas density was found. Gas density did not impact on the measured FI. In prone and supine position the FI of the anterior and posterior lung were significantly different with the FI of the anterior lung > 1 and the FI of the posterior lung was < 1 for all gas mixtures (*P <* 0.01). The differences between the anterior and posterior lung were greater in prone compared to supine. In lateral position similarly the FI of the left and the right lung were significantly different with the FI of the left lung > 1 and the FI of the right lung the FI < 1, independent of the gas mixture used (*P <* 0.05).

## Discussion

The effect of gas density on overall pulmonary function has been investigated in the past and has lead to changes in clinical practice, *e.g.*: the use of helium-oxygen mixtures to treat patients with severe upper airway obstruction or asthma [26,27]. With the introduction of lung imaging techniques such as He3MRI, the impact of gas properties on ventilation distribution needs to be addressed. To maximize the signal to noise ratio, pure helium is generally used as the breathing gas [28]. Interpretation of the images obtained assumes that the distribution of helium is the same as that of air. This may not be the case as properties such as density and viscosity impact on both convection- and diffusion-dependent ventilation distribution [29].

### Spatial ventilation distribution

Inhaled gas density did not have an effect on spatial ventilation distribution. Similar to our previous experimental study in rats, the non-dependent lung was better ventilated than the dependent lung [11] independent of gas density. We further found that regional amplitudes and the geometric centre were dependent on body position but not gas density. The GI was independent of gas density or body position. Verbanck et al. showed minimal convection dependent ventilation inhomogeneity in the rat [10,30]. This is consistent with the results of the current study, where no differences in convection dependent ventilation distribution were found using gas mixtures with different density.

### Regional (temporal) filling characteristics

EIT gives temporal data on regional filling [25]. In our study we found an anatomically dependent regional filling of the lung. In supine and prone positions the posterior lung showed for all gas mixtures a filling index (FI) of less than 1, which indicates a greater rate of filling than the rest of the lung at the beginning of the inspiration. The anterior lung showed the opposite behaviour with a FI > 1, which indicates a slower filling than the rest of the lung at the beginning of the inspiration. In lateral positions the rate of filling at the beginning of the inspiration was always greater in the left lung and again independent of gas density. These differences between the anterior to posterior FI measured were greater in prone than in supine for all gas mixtures, and similar in left lateral position the difference of the FI between the right and left lung was greater in left lateral position (Figure 4). The arbitrary separation of the image into anterior, posterior, left and right segments may affect the calculation of the filling index. The heart shifts towards the dependent lung under gravity. In supine position the heart is suspended from the sternum, whereas in prone position (the natural posture of the rat) the heart is resting on the sternum. Thus the contribution of the filling of the anterior lung will be less in supine position. In lateral position, as previously shown [11], these findings are mostly explained by anatomical characteristics of the rat lung with the right lung larger than the left and extending across the midline into the left side of the chest.

### Limitations

Anatomical differences between the rat and human lung may limit the applicability of the study. However, the rat is a common model for the development of lung imaging techniques such as He3 MRI, and the effect of gas properties on ventilation distribution need to be known.

Most tracer gas inhalation imaging techniques investigate steady-state ventilation images that are obtained after several inhalation cycles of the tracer gas [3]. The images of these techniques are dependent on both convention and diffusion of the inhaled gas mixture Studies [1,10,31] have shown that there is a complex interaction between convection and diffusion dependent ventilation inhomogeneity. EIT measurements are based on impedance changes within the chest [32]. During tidal breathing the majority of the impedance changes occur in the large and small airways as a result of mostly convection dependent volume changes. The alveolar volume in healthy subjects changes minimally during regular breathing and only minimal changes in impedance occur in the alveolar regions [33]. Hence EIT primarily detects changes in ventilation distribution that are convection dependent.

## Conclusion

Gas density independent distribution of regional impedance amplitudes is an important finding for future application of lung imaging techniques such as hyperpolarised helium MRI. We have shown that the gas density of the inhaled tracer gases is not a potential important confounder in the convection dependent distribution of the tracer gas. Direct comparison of EIT and tracer gas imaging techniques is required.

## Competing interests

The authors declare that they have no competing interests.

## Authors’ contributions

JF and AS conceived the study. KD, MF and GC carried out the laboratory work and data analysis. KD and AS drafted the manuscript, to which all authors contributed and approved the final version.

## Funding

This study was funded by the National Health and Medical Research Council.
